# Efficacy of functional electrical stimulation in rehabilitating patients with foot drop symptoms after stroke and its correlation with somatosensory evoked potentials—a crossover randomised controlled trial

**DOI:** 10.1007/s10072-022-06561-3

**Published:** 2022-12-21

**Authors:** Marko Mijic, Benedikt Schoser, Peter Young

**Affiliations:** 1 Department of Neurology, Friedrich-Baur-Institute, Klinikum der Universität, Ludwig-Maximilians-University, Munich, Germany; 2 Clinic for Neurology, Medical Park, Reithof 1, 83075 Bad Feilnbach, Germany

**Keywords:** Functional electrical stimulation, Neuroprostheses, Somatosensory evoked potentials, Stroke rehabilitation, Sensory and motor recovery

## Abstract

**Objective:**

The connectivity between somatosensory evoked potentials (SEPs) and cortical plasticity remains elusive due to a lack of supporting data. This study investigates changes in pathological latencies and amplitudes of SEPs caused by an acute stroke after 2 weeks of rehabilitation with functional electrical stimulation (FES). Furthermore, changes in SEPs and the efficacy of FES against foot drop (FD) stroke symptoms were correlated using the 10-m walk test and foot–ankle strength.

**Methods:**

A randomised controlled two-period crossover design plus a control group (group C) was designed. Group A (*n* = 16) was directly treated with FES, while group B (*n* = 16) was treated after 2 weeks. The untreated control group of 20 healthy adults underwent repeated SEP measurements for evaluation only.

**Results:**

The repeated-measures ANOVA showed a decrease in tibial nerve (TN) P40 and N50 latencies in group A after the intervention, followed by a decline in non-paretic TN SEP in latency N50 (*p* < 0.05). Moreover, compared to groups B and C from baseline to 4 weeks, group A showed a decrease in paretic TN latency P40 and N50 (*p* < 0.05). An increase in FD strength and a reduction in step cadence in group B (*p* < 0.05) and a positive tendency in FD strength (*p* = 0.12) and step cadence (*p* = 0.08) in group A were observed after the treatment time. The data showed a moderate (*r* = 0.50–0.70) correlation between non-paretic TN latency N50 and step cadence in groups A and B after the intervention time.

**Conclusion:**

The FES intervention modified the pathological gait in association with improved SEP afferent feedback.

Registered on 25 February 2021 on ClinicalTrials.gov under identifier number: NCT04767360.

**Supplementary information:**

The online version contains supplementary material available at 10.1007/s10072-022-06561-3.

## Introduction

In our previous systematic review [1], we searched for studies using somatosensory evoked potentials (SEPs) to demonstrate cortical sensory changes in healthy subjects or to estimate cortical plasticity and rehabilitation prognosis in stroke patients after peripheral electrical stimulation (PES) intervention.

SEPs are time-locked potentials evoked by electric stimulation of the sensory or mixed peripheral nerves and recorded along the large-fibre somatosensory (dorsal column–medial lemniscus) pathway [2]. Measures of SEP latencies, thresholds and evoked responses at high stimulator intensities have the highest reliability and require the smallest sample sizes to power a study adequately [3].

PES is a rehabilitative technology that uses electrical currents to the peripheral nerves. It has been proposed that somatosensory stimulation in the form of electromyographically triggered neuromuscular electrical stimulation to the peripheral nerve can influence functional measures of motor performance in stroke patients and produce changes in cortical excitability [4, 5]. The literature offers multiple terms for PES: transcutaneous electric nerve stimulation [6–9], functional electrical stimulation (FES) [10–14], cutaneous electrical stimulation [15], somatosensory stimulation [16], neuromuscular electrical stimulation [4, 17] or a combination of the terms ‘percutaneous’ and ‘neuromodulation’. Sheffler and Chae [14] gave serious consideration to the use of electrical stimulation for motor relearning under the term ‘functional electrical stimulation’ instead of PES. They described three types of electrical stimulation available for motor learning: cyclic functional electrical stimulation (FES), electromyography- or biofeedback-mediated FES, and application of neuroprostheses. In the first case, the patient is a passive participant in the FES training, and no cognitive investment is necessary. The second type of exercise combines afferent feedback information with FES-induced repetitive movements. During training with a neuroprosthesis, functional tasks can be performed [5]. All interventions use the same technique, placing surface electrodes on the skin overlaying sensory-motor nerve structures, establishing an electric field between two electrodes through a medium containing dissolved ions and generating a current in the tissue. Hereinafter, only the term FES will be used.

Our previous review found a correlation between different FES types and changes in SEP components. However, verifying the degree of correlation between SEPs and cortical plasticity was not possible. An interesting finding was that the stroke studies involving FES that initiated a voluntary contraction used for a specific movement or task indicated a positive relationship and correlation with assessments of motor function [13, 15, 17, 18]. Moreover, in stroke studies, patients suffering from foot drop (FD) [13, 15] caused by subcortical or cortical lesions showed remarkable increases in walking speed, endurance and coordination after the application FES. FD is a common gait impairment derived from this pathology and consists of paralysis or significant weakness of the ankle dorsiflexor muscles. It is characterised by the inability to achieve adequate dorsiflexion to obtain a sufficient distance from the ground during the swing phase of gait [19]. FD is also characterised by uncontrolled plantar flexion immediately after initial contact [20].

Therefore, for this trial, the decision was made to apply neuroprosthetic FES using the L300 Go system [21] in patients suffering from post-stroke FD caused by an acute subcortical and cortical lesion. The L300 Go system was designed to improve gait in people suffering from FD and knee flexion or extension in individuals with muscle weakness caused by stroke. The system communicates wirelessly to deliver electrical pulses over the common peroneal nerve and to the motor point of the anterior tibialis muscle, causing ankle dorsiflexion in the swing phase of gait to prevent FD. The effectiveness of eliciting muscle contraction force depends on the electrical stimulation signal’s amplitude, duration, frequency and waveform. The external pulse generator can activate one or two stimulation channels, depending on the cuff type and electrode pre-set [21].

The degree of correlation between SEP and cortical plasticity remained elusive due to a lack of supporting data in previous studies. However, neurophysiological measures may also have predictive value. According to Feys et al. [22], the combination of the motor score and somatosensory evoked potentials (SEPs) is best able to predict an outcome, especially in the acute phase of stroke, since neurophysiological measures alone are of limited value in predicting a long-term effect. Moreover, predictive accuracy is substantially improved by using electric measures and clinical variables [22]. Previous research by Kato et al. [23], who examined the SEPs of the median nerve (MN) and tibial nerve (TN) in patients with haemorrhagic brain lesions, reported that 60 out of 65 arms (92.3%) and 50 out of 62 legs (80.6%) showed abnormalities in SEPs [23]. To overcome this hurdle, we performed a short-term crossover randomised controlled intervention using FES and SEPs in patients with acute stroke.

Our primary aim was to detect changes in pathological latencies and amplitudes of SEPs caused by an acute subcortical and cortical poststroke lesion after a 2-week FES neuroprosthesis FD treatment by observing two stroke groups during intervention and non-intervention times. In addition, an untreated control group of healthy adults underwent repeated SEP measurements for evaluation only.

The second aim was to correlate the detected SEP changes with the efficacy of a 2-week FES neuroprosthesis intervention in improving FD weakness as measured by a 10-m walking test and the strength of dorsal flexion using manual muscle testing grading systems (MRC) [24] between the two stroke groups. Furthermore, we correlated SEP changes with the results of the 10-m walking test and the strength of dorsal flexion MCR between stroke groups.

## Methods

### Study approval and the study protocol

A randomised clinical sequential two-period crossover-design protocol was constructed. The PICO [25] model was implemented to answer the primary clinical question (online data 1) https://osf.io/3dsu6. The study protocol was registered at ClinicalTrials.gov under the identifier number NCT04767360 on 25 February 2021, and data collection ended on 19 October 2021. All participants signed written consent forms. The study was approved by the ethics committee of the Ludwig-Maximilians-Universität München. All examinations were performed as part of a 4-week inpatient neurological rehabilitation in the neurological clinic at Medical Park, Reithof Park, Bad Feilnbach, Germany. The sample size was calculated in a pilot study in a homogeneous population of patients with nearly identical impairments.

Furthermore, 20 healthy subjects underwent a non-interventional second measurement 4 weeks after baseline to determine the maximum fluctuation in the latencies and amplitudes of subcortical and cortical components and in statistical methods for evaluating SEP differences between stroke groups. Thirty-two participants with acute stroke were registered. One patient was excluded due to a repeat stroke, and one healthy volunteer was excluded because of exceeding the timeframe between SEP assessments. Each patient was randomised using https://www.random.org and then began two separate consecutive treatment periods. For the first 2 weeks, group A was directly treated with FES, while group B was treated without FES. After this first 2-week period, group A and group B switched. Group C (healthy participants) received no intervention during the treatment periods. Initially, the ethics commission did not approve the randomised trial with only an intervention group and a control stroke group; therefore, the decision was made to design a crossover study, including a third group with healthy volunteers.

### Patients

The patients were diagnosed with either ischaemic or haemorrhagic stroke within the past 6 months. In this text, the term ‘stroke’ is used for both ischaemic and haemorrhagic stroke. Stroke was confirmed by cerebral computed tomography or magnetic resonance imaging. The inclusion and exclusion criteria were according to the study by Stein et al. [26] and were adapted for our setting. The inclusion criteria for patients were as follows: (1) adults between the ages of 18 and 75 years; (2) inadequate ankle dorsiflexion during the swing phase of gait; (3) inadequate limb clearance as a result of this inadequate ankle dorsiflexion; (4) medically stable condition for at least 1 week following the last episode of stroke; (5) medical clearance to participate with the expectation that current medication can be maintained without change for the next 4 weeks; (6) adequate minimal stability at the ankle during stance with stimulation; (7) adequate cognitive and communication function to give informed consent; (8) the ability to understand the training instructions, use the device, and give adequate feedback; (9) the ability to walk at least 10 m with or without an assistive device. The exclusion criteria eliminated patients with (1) lower motor neuron injury; (2) severe cardiac diseases such as myocardial infarction, congestive heart failure or the need for a demand pacemaker; (3) other electrical stimulation devices in use; (4) hip or knee prostheses made of metal on the lower limb; and (5) epilepsy, autoimmune diseases or tumours.

### Outcomes

The baseline information included age, sex, time of onset, time since stroke and side of stroke lesion. The duration of the entire study examinations was 30 min (including inclusion questionnaires and functional assessments). Sufficient breaks were given between the motor tests. The following motor practical assessments were carried out: ankle dorsiflexion strength measured by MRC classification of muscle imbalance patterns and force produced by voluntary contractions and a 10-m walking test as quickly as possible. The MRC grading system provides the following grades: (0) the ankle is paralysed; (1) only a trace or flicker of muscle contraction is seen or felt; (2) muscle movement is possible with gravity eliminated; (3) muscle movement is possible against gravity; (4) muscle strength is reduced, but movement against resistance is possible; (5) the ankle has normal strength. The 10-m walking test was performed without the FES, and the patient was allowed to use a cane or other walking aid. The same assistive device was used in all sets.

Pedometer records of the steps made with FES were saved for further assessment at the end of a 2-week treatment from the L300 Go System. All data, including SEP, were assessed at baseline, week 2 pre- or post-intervention, and from baseline to week 4. The evaluation of SEP, the 10-m walking test and the measurement of ankle dorsiflexion strength by MRC was made by clinical experts blinded to participants’ clinical information.

### Electrophysiological assessment

The neurological assessment SEPs were recorded from the bilateral MN and TN using a Neurowerk EMG two-channel device by the methods described by Muzyka and Estephan [2]. The standard values for MN and TN were determined for 30 healthy subjects before the start of the study.

The latencies of several components for the MN (N13, N20, P25) and TN (N35, P40, N50) were evaluated, together with central nerve conduction in the MN (P25–N13) and TN (N50–N35). Furthermore, the amplitudes of N20/P25 in the MN and N35/P40 in TN were measured. A detailed description of the SEP measurement process and the measured TN/MN components of the primary somatosensory complex can be found (online data 2) https://osf.io/n3ckg. The SEP was measured at least 1 h after FES therapy.

### Intervention

The intervention group received FES therapy of at least 30 min five times per week. A trained physiotherapist performed the FES therapy and assessments. The following measurements were performed under FES treatment: gait training, balance exercises, and strength exercises while standing and walking, including staircase walking. Groups A and B received the same standard therapies: physiotherapy, resistance training therapy and treadmill therapy. The muscle contraction force elicited by FES was adjusted by setting the amplitude, duration, frequency and waveform of the electrical stimulation signal. Each parameter was set individually depending on the gait parameter quality (Table 1).

**Table 1:** FES stimulation parameters* (Bioness L300 Go)

	Group A	Group B
Intensity (strength of stimulation)	42.71 mA ± 18.78*	41.84 mA ± 15.53*
Phase duration (length of time of the pulse)	200 or 300 μs	200 or 300 μs
Pulse rate (frequency of stimulation)	30 to 50 Hz, in 5-Hz steps	30 to 50 Hz, in 5-Hz steps
Type of electrode: *quick fit* (one channel) or *steering* (two channel) stimulation	14 patients/quick fit 2 patients/steering	13 patients/quick fit 2 patients/steering
Waveform	Symmetric	Symmetric

*Values are given as the mean values with standard deviations

### Statistical analyses

Statistical analyses were performed using STATISTICA software (version 10; Tulsa, OK, USA). All data were normally distributed, as evaluated using the Shapiro‒Wilks test. To compare pre-test–post-test improvement with the two therapy protocols at each follow-up point between the groups, group (2 levels) × time (2 levels) repeated-measures ANOVA was used. We used dependent *t* tests to evaluate pre-test–post-test improvement within groups, and the Pearson correlation coefficient was computed to assess the linear relationship between variables. A positive or negative correlation coefficient was considered high if its absolute value was between 0.70 and 0.90, moderate if its absolute value was between 0.50 and 0.70, and low if its absolute value was below 0.50 [27]. A* p* value of ≤ 0.05 was considered statistically significant for all analyses, and the 95% confidence interval for the mean difference was calculated for each dependent variable. No significant difference among groups was found in sex, age, time of onset or time since stroke (Table 2).

**Table 2:** Patient characteristics and descriptive data

	Group A	Group B
Age, years	61.50 ± 9.01*	61.60 ± 8.96*
Sex	12 males, 4 females	12 males, 3 females
Type of stroke	3 × Pontine infarct, left 1 × Pontine infarct, right 4 × Middle cerebral artery infarct, left 1 × Bihemispheric middle cerebral artery infarct media 1 × Capsula interna infarct, left 1 × Incomplete medial infarct, right 1 × Cortical ischaemia (no further diagnosis description) 1 × Precentral gyrus infarct 1 × Bilateral cerebral haemorrhage 1 × Capsula interna haemorrhage, left 1 × Cerebellar haemorrhage, right	6 × Pontine infarct, left 1 × Pontine infarct, right 4 × Middle cerebral artery infarct, left 1 × Middle cerebral artery infarct, right 1 × Precentral gyrus infarct, right 1 × Anterior choroidal artery infarct, left 1 × Frontoparietal cerebral haemorrhage, left
Time since stroke, days	25.33 ± 14.02*	31.93 ± 15.76*
Hemiparetic side	15 Rt, 1 Lt	15 Rt
Dominant hand affected	13	11
Steps taken with FES	28,849 ± 23,056	25,657 ± 22,753

*Values are given as the mean values with standard deviations

## Results

**Fig. 1:**
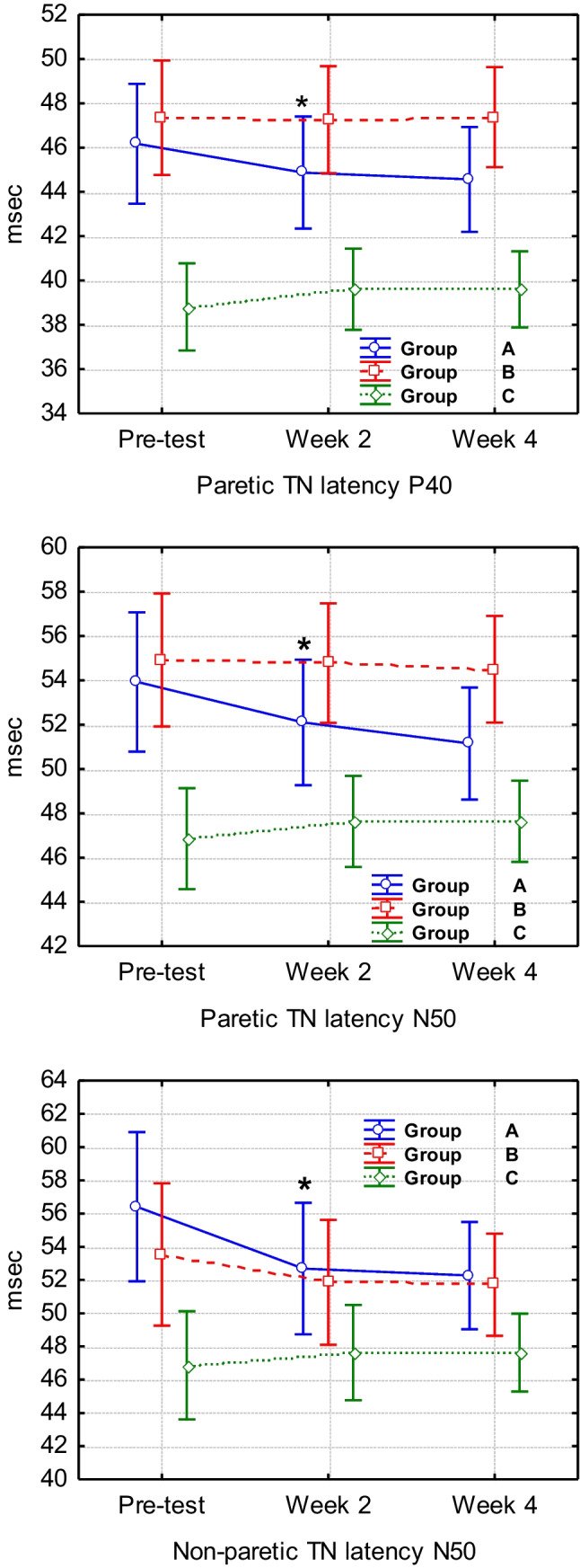
Variation of TN SEP latency: P40 and N50 of the paretic limb and N50 of the non-paretic limb. Vertical bars denote 95% confidence intervals for means. *Comparison of group A to groups B and C from baseline to 2 weeks: decrease in paretic TN P40 latency: *F*(2, 37) = 7.70, *p* = 0.001; decrease in paretic TN N50 latency: *F*(2, 37) = 3,1937, *p* = 0.05254; and decrease of non-paretic TN latency N50: *F*(2, 37) = 8.02, *p* = 0.001

Repeated-measures ANOVA found significant differences between group A and groups B and C (healthy probands) in the changes from baseline to the 2-week examination, consisting of decreased latencies of P40 [*F*(2, 37) = 7.70, *p* = 0.001] and N50 [*F*(2, 37) = 3.19, *p* = 0.052] as well as central nerve conduction velocity [N50–N35: *F*(2, 37) = 3.35, *p* = 0.045] on the affected side. However, in the same period, there were also significant decreases in group A compared to groups B and C in the N50 latency of the non-paretic TN SEP [*F*(2, 37) = 8.02, *p* = 0.001], and as a result of this change, a decrease in central nerve conduction velocity at N50–N35 [*F*(2, 37) = 4.21, *p* = 0.022] was observed. No increase was found in the N35–P40 amplitude of the paretic TN [*F*(2, 37) = 1.88, *p* = 0.166] in the first 2 weeks in group A, B or C. Furthermore, the changes in the paretic TN between 2 and 4 weeks at each of the analysed latencies and amplitudes did not differ among groups A, B and C (*p* > 0.05). An interesting difference was found between group A and groups B and C from baseline to 4 weeks in the decreases in P40 [*F*(2, 37) = 4.71, *p* = 0.014] and N50 [*F*(2, 37) = 3.96, *p* = 0.027] latency in the paretic TN. Important statistically significant changes in the peak latency of SEPs between group A, group B and group C can be found in Fig. 1, and the remaining differences in the SEP variables can be found in Supplementary material 1.

When we examined only group A to group B between baseline and week 2 with *F-*tests of the N35, P40 and N50 latency, N50–N35 central nerve conduction velocity, and N35–P40 amplitude values of the paretic TN, significant changes could not be found (*p* > 0.05). However, positive tendencies were found in the P40 [*F*(1, 19) = 2.63, *p* = 0.120] latency value and central nerve conduction velocity N50–N35 [*F*(1, 19) = 2.16, *p* = 0.157]. In addition, FES was not found to improve pathological latencies or amplitudes in any observed segment between group B and group A between week 2 and week 4 (*p* > 0.05). However, a positive tendency in N35–P40 amplitude could be observed [*F*(1, 19) = 2.83, *p* = 0.108].

**Fig. 2:**
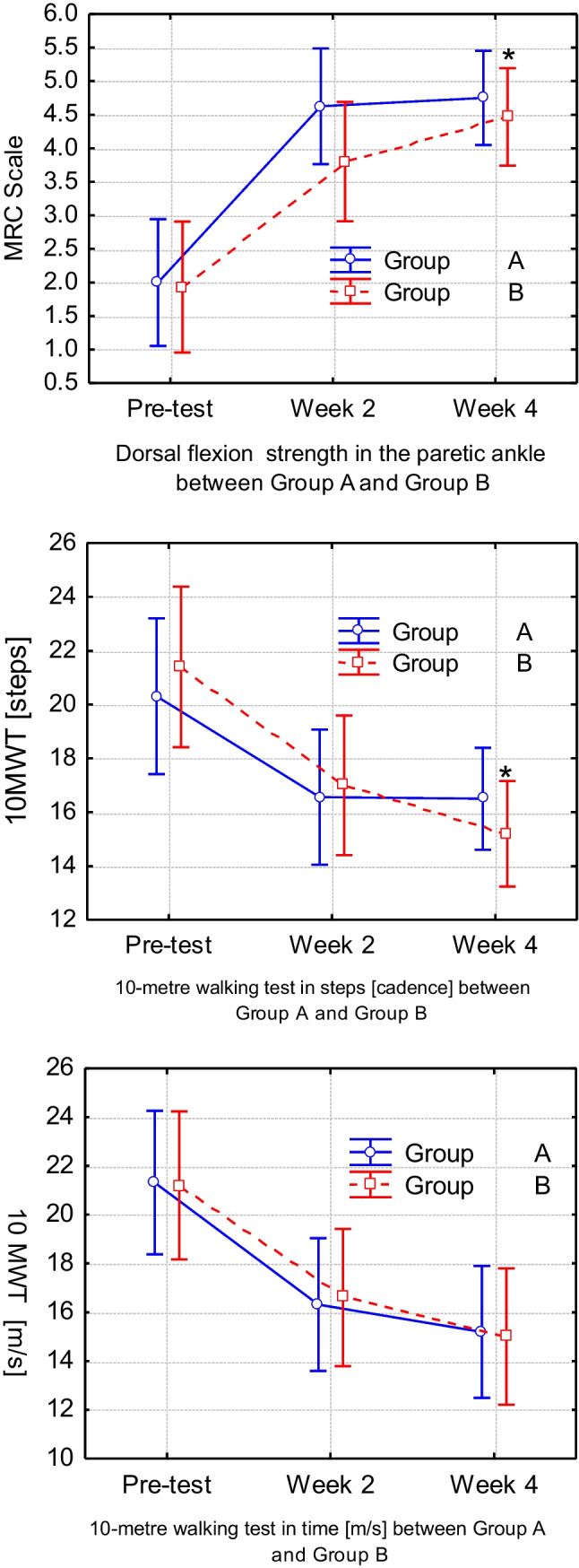
Variation of the motor skill parameters: FD strength and 10-m walk test results (number of steps and time). Vertical bars denote 0.95 confidence intervals for means. *Significant difference in the pre- to post-intervention increase in strength (MRC) in the paretic ankle in dorsal flexion between group B and group A, weeks 2 and 4: [*F*(1, 29) = 4.36, *p* = 0.045] and in steps [cadence] decrease between group B and group A between weeks 2 and 4: [*F*(1, 29) = 4.79, *p* = 0.036]. No effects of FES in 10-m walking test time [s] between groups A and B (*p* > 0.05)

The pre-intervention–post-intervention strength increase in dorsiflexion of the paretic foot ankle between weeks 2 and 4, as measured by MRC classification, significantly differed in group B compared to group A by repeated-measures ANOVA [*F*(1, 29) = 4.36, *p* = 0.045]. Moreover, a positive tendency was found in group A compared to group B between baseline and 2 weeks after the intervention [*F*(1, 29) = 2.50, *p* = 0.124]. *F*-Statistics showed no effects of FES on time or steps in the 10-m walking test between groups A and B in the first 2 weeks (*p* > 0.05). However, a difference was found in the step cadence decrease between group B and group A between weeks 2 and 4 [*F*(1, 29) = 4.79, *p* = 0.036], and there was a positive tendency from baseline to 4 weeks between group A and B [*F*(1, 29) = 3.10, *p* = 0.08]. Groups A and B did not show any changes in the 10-m walking test time in any measurement period. The significant differences between groups A and B from baseline to week 2 and week 4 are shown in Fig. 2.

An essential factor to mention is that a dependent *t* test within groups showed significant changes in 10-m walking test in both groups in the first 2 weeks, in time as well as in cadence (*p* < 0.05); by contrast, in weeks 2 to 4, those differences were not found. Nevertheless, the dependent *t* test shows a remarkable increase in strength within both groups in the first 2 weeks of inpatient intervention (*p* < 0.05) compared to weeks 2 to 4, when this difference was not found (*p* > 0.05).

Clinical neurophysiologists were initially unable to evaluate TN SEPs of ten patients and MN SEPs in two patients because SEP amplitudes were absent due to subcortical or cortical lesions. Those patients were excluded from the calculations of the SEP *F-*statistics and correlation data. Four patients recovered cortical evoked peaks that could not be evaluated initially. None of the patients without cortical SEP peaks showed complete recovery of MRC foot drop. Five patients showed reduced foot drop MRC weakness even though TN SEP on the paretic side indicated an increase in TN cortical latency. Two patients initially indicated MRC weakness in the non-paretic (ipsilesional) foot and ankle in dorsiflexion/plantar flexion.

A moderate correlation from baseline to week 2 between the variables [N50 TN non-paretic latency] and [10-m walking cadence] in group A, *r*(10) = 0.65, *p* < 0.05, was found. In addition, a moderate correlation was observed in groups A and B at baseline to week 4 between the variables [N50 TN non-paretic latency] and [10-m walking steps], *r*(21) = 0.52, *p* < 0.05. A correlation matrix with SEP variables and FD weakness parameters can be found in Supplementary material 2.

## Discussion

FES is a standard inpatient neurological intervention for stroke patients with FD. Due to this ethical consideration, denying patients the right to be involved in FES therapy was not an option. Therefore, the decision was made to construct a crossover design. Including the third group with healthy volunteers was required for three reasons. First, finding the maximal relative difference between healthy individuals for the SEP amplitude and latencies between 4 weeks of measurements is necessary. This gives us information about the standard deviation and sensibility of SEP measurement. Second, the third group increased the number of study participants with minimal detected SEP variation, increasing the validity of the results. Finally, no previous study compared stroke patients and healthy individuals by measuring SEP differences.

Following the interventions, SEP changes found in group A (time since stroke, 25.33 ± 14.02 days) support the hypothesis that post-stroke functional recovery occurred primarily over the first 30 days, with moderate recovery continuing for 30–90 days [28]. However, we are aware of confounding bias created due to spontaneous recovery and endogenous plasticity, which occurs most intensively in the first weeks after stroke [29, 30]. Moreover, few human studies have explicitly sought to test whether intense training early after stroke can augment spontaneous biological recovery [29]. Some retrospective studies on stroke populations suggest that early initiation of rehabilitation is associated with better outcomes [31–33]. These studies had similar confounding to this trial because patients involved in rehabilitation are generally sicker and more severely affected and thus less likely to improve regardless of the timing of care [34]. The time from stroke to rehabilitation inclusion in our study for group B was 31.93 ± 15.76 days, which might mean that the intervention is too late [35]. These results can be substantiated with a dependent *t* test within groups, which showed significant changes in 10-m walking time in both groups in the first 2 weeks, in time and steps cadence (*p* < 0.05) as well as in increase of strength, compared to weeks 2 to 4, where those differences were not found (*p* > 0.05).

LL hemiparesis was found independent of whether the lesions were cortical, subcortical or in the brainstem. Nevertheless, the pathology of SEP in the primary cortical somatosensory complex of the paretic foot can be observed independent of the height of the lesion within the brain. This phenotype was previously explained by Krakauer and Carmichael [29] and Kato et al. [23]. In our case, 11 patients had left/right pons infarcts, and 11 had left/right middle cerebral artery infarcts. It seems that the SEP recordings along the projection fibre pathway (dorsal column–medial lemniscus) allow the identification of abnormalities independent of the lesion location. The researchers considered the spontaneous physical recovery process, which is far more prominent in the acute phase of stroke than in the chronic phase [29], and that found minimal variance in paretic TN cortical peaks P40 and N50 could be attributed to those changes. On the other hand, detected changes in TN SEP could not be found in MN SEP, even though 19 patients showed at least one pathological latency (N20, P25) or amplitude (N20/P25) in the primary somatosensory cortex of the UL. Moreover, those variations could not be observed in the norm data obtained by healthy subjects and maximal deviation given by Muzyka and Estephan [2]. Independently, all patients demonstrated at least one form of subcortical or cortical reorganisation upstream of the TN, reflected in latency or amplitude, after treatment with FES.

No patient showed a loss or decrease in TN cortical peaks after the FES intervention, which could be generated as a result of aggravation or as already described in studies [7, 36, 37] in which the use of FES decreased cortical somatosensory amplitudes. Analysing common values of healthy subjects with stroke patients, all patients showed at least one pathology in loss of cortical waves, delay in peak latencies (N35/P40/N50) or reduction in amplitude (N35/P40). These results correspond to the outcome obtained in the study from Kato et al. [23]. Finally, the lack of SEP changes by examining group A to group B without healthy subjects is probably caused by the number of cases and the short assessment timeframe. However, a positive tendency seen in several components supports our opinion that there is an influence of FES on SEP.

The dependent *t* test found improvements in both cadence and time in the 10-m walking test in both groups during 4-week inpatient neurological rehabilitation. Moreover, the dependent *t* test showed a remarkable increase in strength in both groups. These results were expected because, in addition to FES, all patients received other standard therapies as well: physiotherapy, resistance training therapy, treadmill therapy and activities of daily living training. The MRC scale data (Fig. 1) show that group A recovered faster within the first 2 weeks and group B in the second 2 weeks in the paretic feet ankle in dorsal flexion strength. Five patients showed recovery in FD strength measured by MRC classification even though the TN SEPs of the paretic side still indicated an increase in TN cortical latency and amplitude. Moreover, FD was still obtained, and sufficient distance from the ground during the swing phase of gait was clinically not visible. A study by Bao et al. [13] reported that LL strength is necessary but insufficient to produce recovery of voluntary control of coordinated and rhythmic movements in patients poststroke. Recovery of joint mobility and rhythmic movements should not be omitted due to their impact on the recovery of central nervous system motor control function [13]. This statement indicates that reduced coordination and rhythmic foot movement can be more affected and take longer to recover, whereas strength in FD recovers more quickly in the first weeks after stroke. These results are similar to the data found in our study.

Interestingly, group A showed no improvement in steps taken in the 10-m walking test between weeks 2 and 4, during which no intervention was given. This showed that using individual adjusted FES on stroke patients could help reduce this impairment by providing an electrical stimulus in the average gait rhythm pace. Furthermore, a moderate correlation between changes in the variable [N50 TN non-paretic latency] and the variable [10-m walking test in cadence/group A], *r*(10) = 0.65, *p* < 0.05, over intervention time was found. These data, in addition to changes in TN SEP N50 latency on the non-paretic side, could reveal the influence of the ipsilateral side on cortical reorganisation after stroke. Moreover, ipsilateral foot weakness was found in two patients. This phenomenon was already described by Xu et al. [38] on maximal voluntary contraction force in the unaffected side/hand. We remain careful about further statements on the influence and changes found in non-paretic SEP latency N50 since the cortical reference electrode of the TN for the right and left sides is in a single recording location, and lower subcortical changes cannot be evaluated using this measuring method. Last, FD recovery was observed only in patients with retained SEPs. The correlation between SEP and gait parameters after intervention time in both groups demonstrated a relationship between sensory and motor brain areas. It is known that there are substantial anatomical interconnections linking the brain’s motor and somatosensory regions. Cortical motor areas receive direct inputs from the primary and second somatosensory cortex. Conversely, somatosensory areas receive direct cortical inputs from the primary motor cortex, premotor cortex and supplementary motor area [39, 40]. A change in somatosensory function associated with motor learning would seem to be a natural by-product of this anatomical connectivity [39, 40]. The findings in this study suggest that FES may shift the somatosensory response to the brain’s motor areas. On the other hand, it could be hypothesised that SEP can indirectly recognise changes in the brain’s motor area.

Furthermore, SEPs have sufficient sensitivity to detect even the smallest changes in the action potential of cortical neural networks after stroke. They can probably be used to assess the effect of various sensory therapies—cryotherapy, thermotherapy, occupational tactile therapy or robotic tactile therapy—in a direct manner. Nevertheless, it would be essential for future studies to assess the influence of FES using a combination of SEP with different neurophysiological measures, such as transcranial magnetic stimulation [40–42] or laser-evoked potentials [43], which have already shown good prognostic value. There is a reasonable proposition that a combination of neurophysiological measures could provide high correlation in the prediction of upper limb (UL) and lower limb (LL) motor recovery in stroke patients [41, 42, 44, 45].

## Study limitations

The patients and the therapist were unblinded during the therapy. We did not include a group that underwent FES only. However, the data from the MN SEP can be considered compensation from our point of view. Moreover, we had healthy volunteers to minimise this bias. We evaluated outcomes only up to week 4 after the intervention; thus, long-term outcomes are still needed to substantiate our results further. We cannot exclude the possibility of selection bias because we used strength inclusion criteria. High technical performance in carrying out SEP measurements and evaluations is necessary to interpret variability in pathological cortical peaks. Even though clinical experts were masked to participants’ clinical information, additional patient data would reduce the bias caused by manual cortical peaks cursor adjustment.

## Conclusion

After a short-term neuroprosthetic FES intervention, we found an improvement in pathological gait function (time, number of steps and reduction of FD weakness). Correlating with the clinical improvement, changes in afferent SEP feedback were observed. This indicates that FES should be included in the stroke rehabilitation process as early as possible. The SEP measurement procedure is time consuming and susceptible to error, and it requires a highly knowledgeable clinician. Our findings indicate the importance of assessing electrophysiological methods and verifying the application of SEP by stroke patients.

## Supplementary information

Below is the link to the electronic supplementary material.

Supplementary file1 (PDF 104 KB)

Supplementary file2 (PDF 100 KB)

## Data Availability

The datasets generated during and/or analysed during the current study are available from the corresponding author on reasonable request.

## Abbreviations

PES: Peripheral electrical stimulation
FES: Functional electrical stimulation
SEPs: Somatosensory evoked potentials
UL: Upper limb
LL: Lower limb
MN: Median nerve
TN: Tibial nerve
FD: Foot drop
MRC: Manual muscle testing grading systems
