# Supplementary material for: Efficacy of functional electrical stimulation in rehabilitating patients with foot drop symptoms after stroke and its correlation with somatosensory evoked potentials—a crossover randomised controlled trial
Source: Neurol Sci. 2022 Dec 21;44(4):1301–10. doi: 10.1007/s10072-022-06561-3 (PMC10023639; doi:10.1007/s10072-022-06561-3)
Supplement: Supplementary file 1 — Supplementary file1 (PDF 104 KB) [file 10072_2022_6561_MOESM1_ESM.pdf]

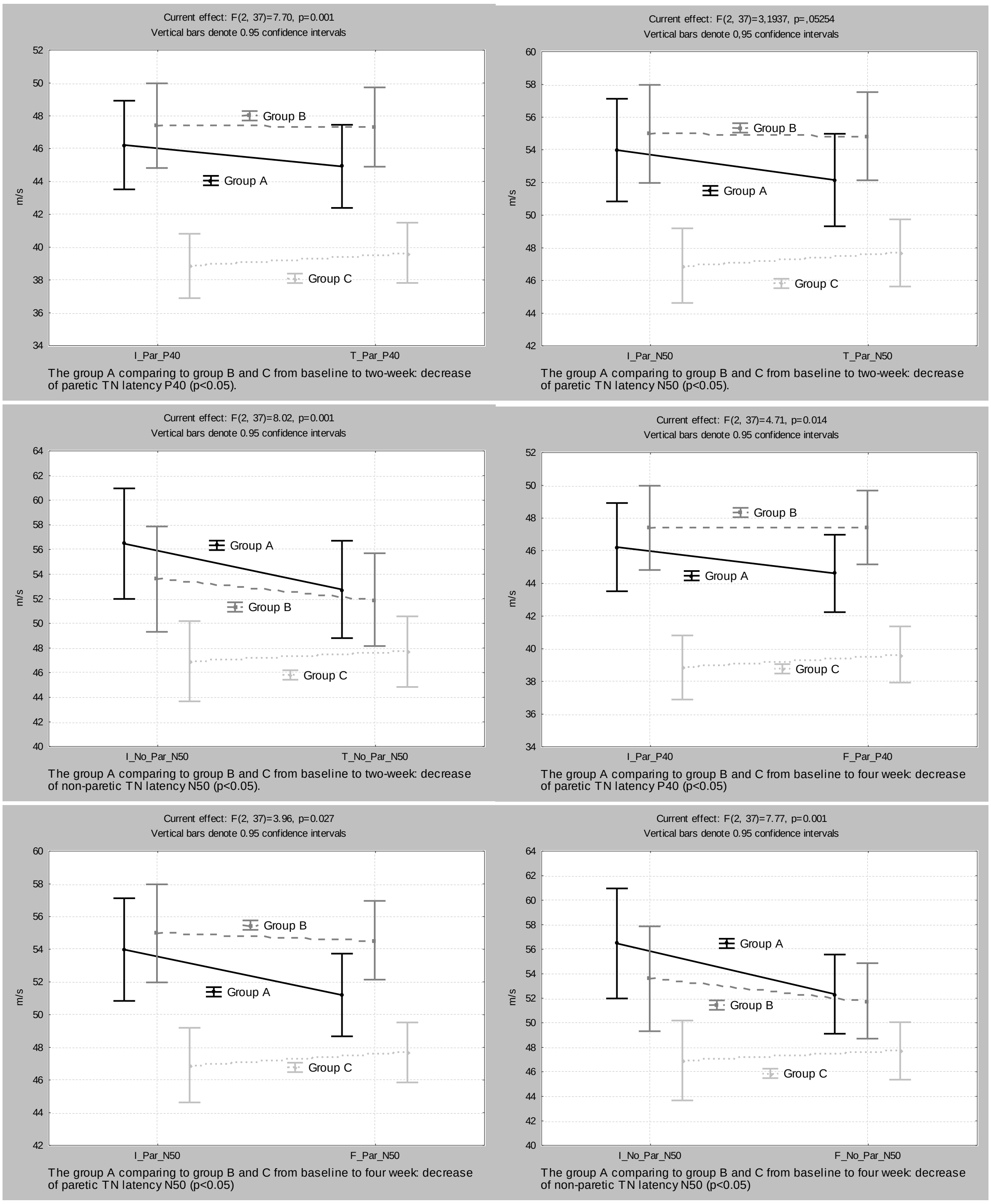

Figure.1. All significant changes in SEP variables; Variation in peaks latency of SEP between Group A, Group B, and Group C. Vertical bars denote 0,95 confidence intervals for means.
