# Supplementary material for: Efficacy of functional electrical stimulation in rehabilitating patients with foot drop symptoms after stroke and its correlation with somatosensory evoked potentials—a crossover randomised controlled trial
Source: Neurol Sci. 2022 Dec 21;44(4):1301–10. doi: 10.1007/s10072-022-06561-3 (PMC10023639; doi:10.1007/s10072-022-06561-3)
Supplement: Supplementary file 2 — Supplementary file2 (PDF 100 KB) [file 10072_2022_6561_MOESM2_ESM.pdf]

Table 1. Correlation matrix with SEP variables and FD weakness parameters for different timeframes:

Correlations matrix between SEP latencies and motor scores in Group A from pre-test to week two

Variable	Marked correlations are significant at p <0,05<br>N=10					
	P40 paretic lower limb	N50 paretic lower limb	N50 non-paretic lower limb	MRC Scale	10MWT m/s	Steps
P40 paretic lower limb	1.00	**0.81***	**0.73***	-0.07	0.11	0.10
N50 paretic lower limb		1.00	**0.67***	-0.29	0.20	0.23
N50 non-paretic lower limb			1.00	0.17	0.31	**0.65***
MRC Scale				1.00	-0.32	0.04
10MWT m/s					1.00	**0.70***
Steps						1.00

Correlations matrix between SEP latencies and motor scores in Group B from week two to week four

Variable	Marked correlations are significant at p <0,05<br>N=11					
	P40 paretic lower limb	N50 paretic lower limb	N50 non-paretic lower limb	MRC Scale	10MWT m/s	Steps
P40 paretic lower limb	1.00	**0.81***	0.41	- 0.34	0.03	0.05
N50 paretic lower limb		1.00	-0.25	0.39	0.04	-0.18
N50 non-paretic lower limb			1.00	0.23	-0.14	-0.11
MRC Scale				1.00	-0.33	**-0.57***
10MWT m/s					1.00	-0.30
Steps						1.00

Correlations matrix between SEP latencies and motor scores in Group A and Group B from pre-test to week four

Variable	Marked correlations are significant at p <0,05<br>N=21					
	P40 paretic lower limb	N50 paretic lower limb	N50 non-paretic lower limb	MRC Scale	10MWT m/s	Steps
P40 paretic lower limb	1.00	**0 .83***	-0.49	-0.29	-0.05	-0.23
N50 paretic lower limb		1.00	**0.60***	0.25	-0.04	-0.22
N50 non-paretic lower limb			1.00	0.12	-0.11	**0.52***
MRC Scale				1.00	-0.43	0.44
10MWT m/s					1.00	-0.44
Steps						1.00

*The high positive (negative) correlation coefficient was considered if value lies between 0.70 to 0.90, moderate degree value positive (negative) between 0.50 to 0.70 and if the value lies below 0.50 is interpreted as a positive (negative) low correlation (1).

1. Mukaka MM. Statistics corner: A guide to appropriate use of correlation coefficient in medical research. *Malawi Med J*. 2012;24(3):69–71.
